# Trends in quality of life reporting for radical cystectomy and urinary diversion over the last four decades: A systematic review of the literature

**DOI:** 10.1080/2090598X.2019.1600279

**Published:** 2019-04-14

**Authors:** Karan Rangarajan, Bhaskar K. Somani

**Affiliations:** University Hospital Southampton NHS Trust, Southampton, UK

**Keywords:** Quality of life, cystectomy, ileal conduit, neobladder, urinary diversion, review

## Abstract

**Objective**: To report the trends in quality of life (QoL) reporting for radical cystectomy (RC) and urinary diversion (UD) over the last four decades, as RC for bladder cancer is associated with significant morbidity and QoL issues.

**Material and methods**: We searched PubMed, Medical Literature Analysis and Retrieval System Online (MEDLINE), Excerpta Medica dataBASE (EMBASE), Cumulative Index to Nursing and Allied Health Literature (CINAHL), and the Cochrane library for published studies from January 1980 to January 2017 in the English language. We divided the published articles into three time periods: period-1 (1980**–**1997), period-2 (1998–2007) and period-3 (2008–2017).

**Results**: A total of 85 QoL studies (8417 patients) were identified, of which 3347 (39.8%) patients had an ileal conduit (IC), 1078 (12.8%) had a continent UD (CD), 3264 (38.8%) had a neobladder (NB), and in the remaining 728 (8.6%) the type of UD was not specified. Whilst there were 15, 24 and 41 studies in period-1, period-2 and period-3 respectively, two (13%), 20 (83%) and 37 (90%) used a validated QoL tool; and none, six (25%) and 23 (56%) used a urology specific QoL tool during these three time periods. Similarly, the number of prospective studies increased from one (7%) to four (17%) and 14 (34%) in these three time periods. The proportion of reported IC patients reduced from 65% (784 patients) to 36% (899) and 35% (1664) from period-1 to period-3, whereas the proportion of NB patients increased from 4.5% (54) to 44% (1105) and 44% (2105). Over the last few years there have been QoL studies on laparoscopic and robotic IC and NB UDs.

**Conclusion**: Our review suggests an increasing use of validated, bladder cancer-specific questionnaires with UD-specific constructs.

**Abbreviations**: BCI: Bladder Cancer Index; BDI: Beck Depression Inventory; BIS: Body Image Scale; CD: continent urinary diversion; EORTC QLQ-30C: European Organisation for the Research and Treatment of Cancer Quality of Life 30-item core questionnaire; ERAS: enhanced recovery after surgery; FACT(-BL)(-G)(-VCI): Functional Assessment of Cancer Therapy(-Bladder Cancer)(-General)(-Vanderbilt Cystectomy Index); IC: ileal conduit; NB: neobladder; (HR)QoL: (health-related) quality of life; (RA)RC: (robot-assisted) radical cystectomy; SF-36: 36-item short-form health survey; SIP: Sickness Impact Profile; UD: urinary diversion

## Introduction

Radical cystectomy (RC) with urinary diversion (UD) is associated with significant morbidity. Once patients recover from this surgery, quality of life (QoL) becomes an important priority having a significant role in their future psychological and emotional well-being [1–3]. UD impacts QoL and there are different types of UD to choose from, including ileal conduit (IC) to continent cutaneous UD (CD) and neobladder (NB) [3–8].

Measuring QoL can help assess the impact of RC and UD, identify patient preference, help in staff training, and be useful for audit and clinical governance [1]. The choice of UD depends on patient suitability and preference, with a possible surgical bias related to the surgical expertise available in the centre. Whilst enthusiasts favour NB, there is little evidence to support that one UD type is better than another [9–12]. It seems that for now, the choice of UD should be individualised and based on patient counselling and expectations, with an active but unbiased surgical input. Measuring QoL in these patients has changed from self-designed to non-validated and now validated tools, including generic and disease-specific measures. Over time the shift has been to use bladder cancer-specific health-related QoL (HRQoL) tools supplemented by patient-reported outcome measures [8,13–18].

Publication trends reflect clinical practice [19]. Trends in the type of UD offered could help patients in their choice of UD type, improve counselling and allocation of healthcare resources. The QoL aspect seems to be the most important element in UD once patients have recovered from their initial surgery. There is no bibliometric study looking at the publication trends of reporting QoL in UD patients. We therefore assessed the trend in QoL reporting after RC and UD over the last four decades.

## Materials and methods

### Inclusion criteria

1. All studies reporting on QoL after UD, irrespective of the type of UD.
2. Studies published in English language over the last four decades.

### Exclusion criteria

1. Animal studies and case reports.
2. Studies on UD that did not assess QoL.

### Search strategy and study selection

We performed a systematic review of the world literature to identify original studies reporting on QoL in UD. It was carried out using Cochrane and Preferred Reporting Items for Systematic Reviews and Meta-Analyses (PRISMA) methodology.

**Figure 1.**
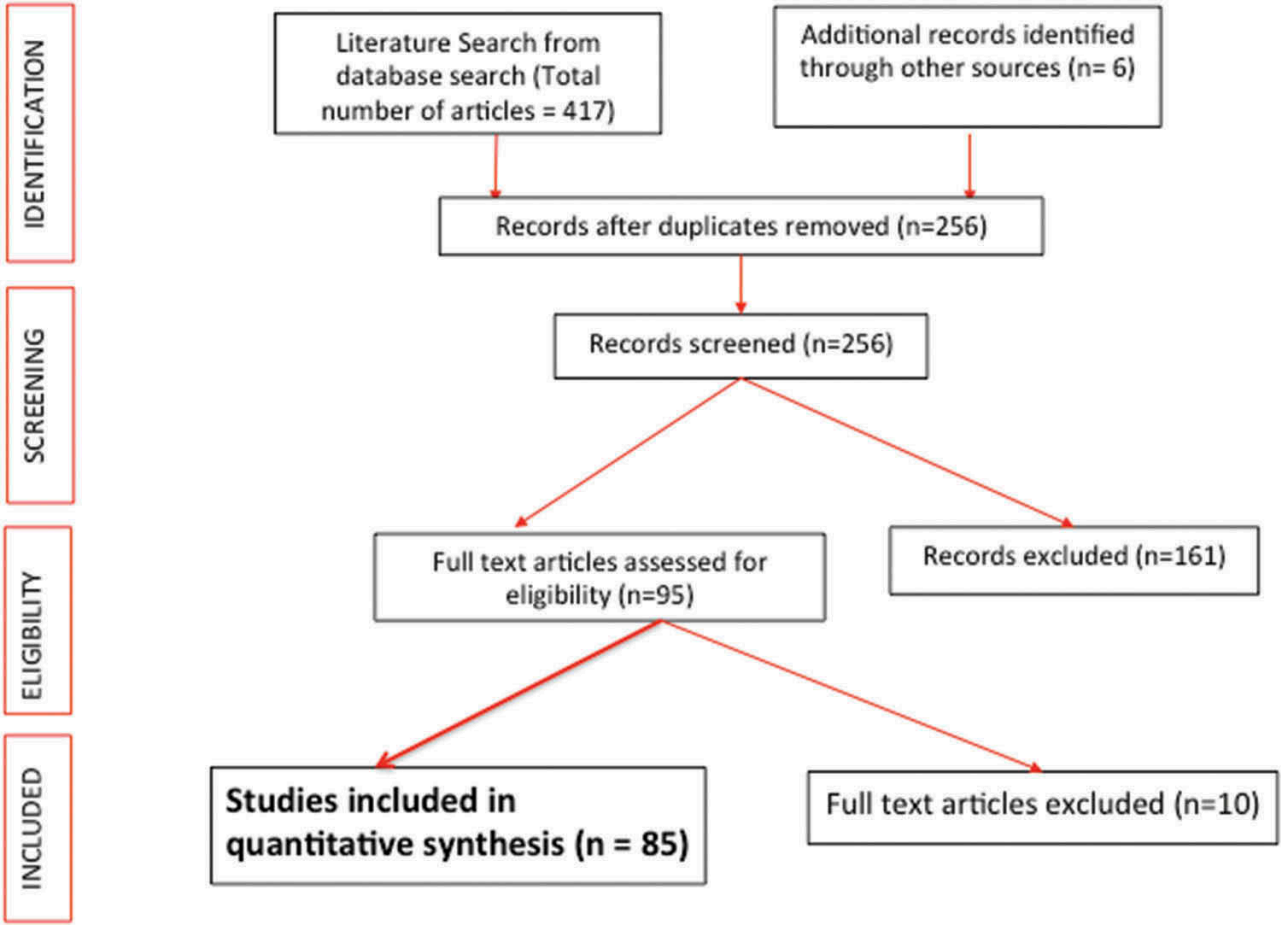
PRISMA flowchart of study inclusion.

We searched PubMed, Medical Literature Analysis and Retrieval System Online (MEDLINE), Excerpta Medica dataBASE (EMBASE), Cumulative Index to Nursing and Allied Health Literature (CINAHL) and the Cochrane library for published studies from January 1980 to January 2017. We used the following search terms ‘urinary diversion’, ‘quality of life’, ‘neobladder’, ‘ileal conduit’, ‘cutaneous diversion’, ‘cystectomy’, ‘health-related quality of life’, and ‘QoL’. All articles from 1980 to 1997, and only articles directly comparing two or more different UD types from 1998 to 2017 were selected for screening. All full-length articles published in the English language were included in the original search and the two reviewers (K.R., B.K.S.) independently identified all studies that fitted the inclusion criteria (Figure 1).

We included all studies where patients underwent UD (1980–1997), and at least two forms of UD were used and QoL was measured using either a validated or non-validated questionnaire (1998–2017). After screening the abstracts (*n* = 295), 85 studies met the inclusion criteria and were included in our present review (Appendix 1). Each of the 85 studies was then assessed in a standardised fashion. The following information was collected for each study: number of patients, type of UD compared, study design, type of instrument used to assess HRQoL outcome (general vs disease-specific and validated vs non-validated), major findings of the study, and any other characteristics unique to the study.

We divided the last four decades into three time periods: period-1 (1980**–**1997), period-2 (1998–2007), and period-3 (2008–2017).

### Primary outcome measures

1. Trends of QoL reporting over the last four decades.
2. Number of studies and type of UD done.

### Secondary outcome measures

1. Geographical variation in the reporting of the QoL studies.
2. Journals which published these QoL studies.

### Data extraction and analysis

Both reviewers (K.R., B.K.S.) independently identified all studies that appeared to fit the inclusion criteria and any disagreement was resolved with mutual consensus. All data were collected in an Excel spreadsheet and then transferred to a Word document. The study was carried out using the Cochrane and PRISMA methodology. Included studies showed a high level of heterogeneity and bias, and data were not suitable for a meta-analysis, and hence have been presented in a descriptive manner.

## Results

Over the last 37 years, a total of 85 post-RC QoL studies (8417 patients) have been reported (Tables 1 and 2). Of these UD patients (within the 85 studies), 3347 (39.8%) had an IC, 1078 (12.8%) had a CD, 3264 (38.8%) had a NB, and in the remaining 728 (8.6%) the type of UD was not specified (Tables 1 and 2). Whilst there were 15, 24 and 41 studies in period-1, period-2, and period-3, respectively, two (13%), 20 (83%) and 37 (90%) used a validated QoL tool; and none, six (25%) and 23 (56%) used a urology specific QoL tool during these three time periods. Similarly, the number of prospective studies increased from one (7%) to four (17%) and 14 (34%) in these three time periods.

**Table 1.** The number of studies and types of UD performed over the last four decades (five studies were overlapping*).

Year	No. of countries (studies*)	No. of patients	Validated scale (urology specific) used, *n*	Prospective/retrospective, *n*	IC/CD/NB/unspecified, *n*	Open/lap or robotic, *n*
1980–1997	6 (15)	1206	2 (0)	1/14	784/368/54/0	1206/0
1998–2007	11 (24)	2464	20 (6)	4/20	899/428/1105/32	2432/0
2008–2017	18 (41)	4747	37 (23)	14/27	1664/282/2105/696	3939/112
Total	35 (80)	8417	59 (29)	19/61	3347/1078/3264/728	7577/112

lap, laparoscopic.

**Table 2.** QoL of the included UD studies over the last four decades (Appendix 1).

	Journal	Author	Country	Year	No. of Patients	Scale used – 1	Scale used 2	IC	CD	NB	CD/NB	Study type	Conclusion on QoL
1	*Br J Urol*	Jones *et al.*	UK	1980	34	Self-designed questionnaire		34				Retro.	Stoma problems
2	*Scand J Urol Nephrol*	Fosså *et al.*	Norway	1987	59	Self – psychological/social issues		59				Retro.	Good QoL
3	*J Urol*	Boyd *et al.*	USA	1987	172	BDI, POMS, physical impact		87	85			Retro.	Preop. counselling important, patients overall satisfied but more for CD
4	*Br J Urol*	Månsson *et al.*	Sweden	1988	60	Self-designed questionnaire		40	20			Retro.	Less stoma problems and more freedom for activities in CD
5	*Scand J Urol Nephrol*	Mommsen *et al.*	Denmark	1989	68	Self-designed questionnaire		68				Retro.	Preop. counselling important but often neglected
6	*Br J Urol*	Chadwick and Stower	UK	1990	41	Interview – appliance management		41				Retro.	83% improved QoL, 90% continue household duty, leakage problem
7	*Scand J Caring Sci*	Månsson *et al.*	Sweden	1991	34	Interview		20	14			Retro.	Sexual problems postop., lack of psychological support from health services – irrespective of UD
8	*Br J Urol*	Nordström *et al.*	Sweden	1992	66	Interview – sexual function		66				Retro.	90% men had erectile dysfunction, 5/6 females had lower sexual activity
9	*Scand J Urol Nephrol*	Nordström *et al.*	Sweden	1992	66	Interview – psychological function		66				Retro.	80% overall good health, 70% unchanged social activity, leak, body image in females
10	*Scand J Urol Nephrol*	Bjerre *et al.*	Denmark	1994	76	Self-designed questionnaire		50	26			Retro.	Global satisfaction high and similar in both groups
11	*Br J Urol*	Bjerre *et al.*	Denmark	1995	67	Interview + questionnaire		29		38		Retro.	High global satisfaction with both UDs, Urinary leak more frequent in NB, but IC patients affected more
12	*J Urol*	Gerharz *et al.*	Germany	1997	192	Self-designed questionnaire		131	61			Retro.	Less stoma problems in CD, overall scores similar
13	*Int J Urol*	Okada *et al.*	Japan	1997	137	Self-designed questionnaire		63	74			Retro.	Less stoma problems in CD, but more night catheterisations, more satisfied patients in CD, counselling/consent
14	*Eur Urol*	Filipas *et al.*	Germany	1997	81	Interview + questionnaire		27	54			Retro.	No difference in global satisfaction and health, UD type must consider psychological and employment status
15	*Scand J Urol Nephrol*	Bjerre *et al.*	Denmark	1997	37	Self-designed questionnaire		20	17			Retro.	No difference in two groups
16	*Br J Urol*	Månsson *et al.*	Sweden	1997	50	SIP	MCT	17	17	16		Pros.	Defensive strategies and philosophical outlook generally did not influence the psychosocial outcome of intervention
17	*Scand J Urol Nephrol*	Bjerre *et al.*	Denmark	1998	76	Self-designed questionnaire		27		49		Retro.	No difference in two groups
18	*Urology*	Weijerman *et al.*	The Netherlands	1998	56	SIP			23	33		Retro.	Overall QoL favourable in both groups
19	*Br J Urol*	Sullivan *et al.*	Canada	1998	86	Urinary symptoms, activity level, overall wellbeing			42	44		Retro.	Good overall QoL, significant effect on sex life, 70% patients had no limit on activities
20	*Br J Urol*	Månsson *et al.*	Sweden	1998	57	Interview + questionnaire	MCT + VAS	17	22	18		Pros.	Patients with wet stoma did not do less well than continent procedures, and the adjustment improved with time
21	*J Urol*	Hart *et al.*	USA	1999	224	4 self-reporting questionnaire		24	93	103		Retro.	Good overall QoL in all groups
22	*Int J Urol*	Kitamura *et al.*	Japan	1999	79	EORTC QLQ-C30	Self-designed questionnaire	36	22	21		Retro.	Little difference in all groups, patients accepted and adapted to present general quality status
23	*Qual Life Res*	Hardt *et al.*	Germany	2000	44	SF-36	FLZM	24	20			Pros.	High global satisfaction with both UDs, 75% would choose same UD again
24	*Ann Surg Oncol*	McGuire *et al.*	USA	2000	92	SF-36		38	16	38		Retro.	IC patients have decreased mental QoL but continent UDs do not, compared to population norms
25	*Urology*	Fujisawa *et al.*	Japan	2000	56	SF-36		20		36		Retro.	No difference in two groups
26	*World J Urol*	Hobisch *et al.*	Austria	2000	102	EORTC QLQ-C30	Self-designed questionnaire	33		69		Retro.	QoL better with NB in all domains
27	*Eur Urol*	Kulaksizoglu *et al.*	Turkey	2002	68	EORTC QLQ-C30	BDI	49	2	15		Pros.	Psychological and HRQoL measures come to baseline values and stabilise after the 12th-month period
28	*BJU Int*	Månsson *et al.*	Sweden	2002	64	FACT-BL	HADS		35	29		Retro.	No difference overall between groups (NB – more incontinence, but better appreciation of appearance and erectile function)
29	*BJU Int*	Hara *et al.*	Japan	2002	85	SF-36		37		48		Retro.	Patients satisfied with overall QoL and health status in both groups
30	J Urol	Dutta *et al.*	USA	2002	72	SF-36	FACT-G	23		49		Retro.	NB marginally better when adjusted for age, stage and sex
31	*Eur Urol*	Henningsohn *et al.*	Sweden	2003	395	Self-designed questionnaire		218	88	89		Retro.	Compromised sexual function main source of distress in RC patients, addressing self-assessed distress may improve patient care
32	*BJU Int*	Protogerou *et al.*	Greece	2004	108	EORTC QLQ-C30	Self-designed questionnaire	58		50		Retro.	QoL same in both groups. Higher emotional function compared to NB population but more urinary + sexual problems
33	*Eur Urol*	Joniau *et al.*	Belgium	2005	58	Self-designed questionnaire				58		Retro.	ONB substitution has acceptable impact on patient’s everyday life.
34	*J Urol*	Yoneda *et al.*	Japan	2005	48	SF-36	FACT-Bl			48		Retro.	No difference in HRQoL between patients and controls
35	*Cancer*	Allareddy *et al.*	USA	2006	82	FACT-BL		56			26	Retro.	No difference in IC vs continent UD; no major difference between non-RC and RC patients
36	*Jpn J Clin Oncol*	Kikuchi *et al.*	Japan	2006	49	FACT-BL		20	14	15		Retro.	QoL – no difference; body image and urinary function affected. 10/13 IC, 7/9 CD, 6/7 NB would choose same operation again
37	*Int J Urol*	Harano *et al.*	Japan	2007	41	SF-36	Urinary continence questionnaire		20	21		Retro.	HRQoL in the NB group and those in the CD group were similar
38	*Cancer*	Gilbert *et al.*	USA	2007	188	BCI		66		122		Retro.	More urinary leak in NB
39	*Acta Med Okayama*	Saika *et al.*	Japan	2007	109	EORTC QLQ-C30	Patient satisfaction	56	31	22		Retro.	No difference in HRQoL, more patients disappointed with NB – preop. counselling
40	*Urology*	Månsson *et al.*	Sweden	2007	61	FACT-BL	HADS			61		Pros.	Swedish men had better FACT-BL and HADS scores, patient assessed outcome differ with different populations
41	*Eur J Surg Oncol*	Autorino *et al.*	Italy	2008	79	SF-36		44		35		Retro.	No significant difference in scores between IC and NB. Compared to control population – physical, social and emotional functioning worse in both IC and NB groups
42	*Urology*	Sogni *et al.*	Italy	2008	85	EORTC QLQ-C30	EORTC QLQ-BLM30	53		32		Retro.	No difference in QoL or complications and survival
43	*BJU Int*	Yuh *et al.*	USA	2009	34	FACT-BL		34				Pros.	Pre- and post-RC QoL, postop. QoL scores similar at 3 months and exceeded baseline at 6 months
44	*Scand J Urol Nephrol*	Frich *et al.*	Norway	2009	72	Self-designed questionnaire		37		35		Retro.	Patients with all UDs rated their QoL as high with no significant difference between them. More patients in NB group experienced practical problems compared to IC. Influence on everyday life was significantly better in favour of IC compared to NB.
45	*Ann R Coll Surg Engl*	Philip *et al.*	UK	2009	52	SF-36		24		28		Retro.	NB patients were younger and more fit. HRQoL was favourable in both UDs, with physical functioning significantly better in NB group. Conclude – body image issues persist although no formal body image measures used.
46	*Urology*	Somani *et al.*	UK	2009	32	SWLS	EORTC QLQ-C30	29		3		Pros.	No difference in scores between IC and NB
47	*BJU Int*	Miyake *et al.*	Japan	2010	80	SF-36				80		Retro.	HRQoL similar except physical health, emotional problems and bodily pain, which were worse in NB patients. No difference between men and women.
48	*J Urol*	Large *et al.*	USA	2010	40	FACT-VCI			19	21		Retro.	Women undergoing RC with ONB vs IP have similar HRQoL outcomes
49	*Urology*	Hedgepeth *et al.*	USA	2010	336	BCI	BIS	85		139		Pros.	Longest F/U – 8 years. Initial worsening of body image in both UDs. Earlier return of body image to baseline for IC, with NB never returning to baseline. Age but not sex associated with body image with older patients having better body image
50	*Int Urol Nephrol*	Vakalopoulos *et al.*	Greece	2011	39	FACT-G	FACT-VCI; BDI; SF-36		14	25		Retro.	Patients with UUC surprisingly presented at least equal QoL than the presumably less debilitating ONB
51	*ISRN Urol*	Erber *et al.*	Germany	2012	301	EORTC QLQ-C30	BLM30	146		115		Retro.	Many arguments in favour of NB rather than IC as the UD of choice.
52	*Urology*	Anderson *et al.*	USA	2012	190	FACT-VCI		70		101		Retro.	Patients with IC had VCI scores that averaged 5 points > than those who had an ONB UD at 1-year postop.
53	*Eur J Surg Oncol*	Miyake *et al.*	Japan	2012	212	SF-36				212		Retro.	HRQoL with NB is generally favourable irrespective of the type of NB
54	*Urology*	Stegemann *et al.*	USA	2012	91	CARE questionnaire		84		6		Pros.	Initial decline in QoL after surgery but approached preoperative baseline levels at ≤90 days
55	*Cir Esp*	Mucciardi *et al.*	Italy	2013	58	EORTC QLQ-C30			58			Retro.	Cutaneous ureterostomy represents a valuable alternative for elderly patients with high surgical risk
56	*Acta Inform Med*	Prcic *et al.*	Bosnia & Hersegovina	2013	106	SIP		66	20	20		Pros.	NB provides significantly better QoL than IC
57	*Korean J Urol*	Shim *et al.*	South Korea	2013	42	K-BIS	Author-constructed questionnaire	13		29		Retro.	NB was associated with significantly better body image than IC
58	*Mol Clin Oncol*	Yang *et al.*	China	2013	82	SF-36	Continence questionnaire (NB group only)		28	54		Pros.	SF-36 scores were significantly greater following NB than non-NB – total health scores were higher
59	*Can J Urol*	Metcalfe *et al.*	Canada	2013	84	FACT-VCI		53		31		Retro.	No statistically significant association between the type of UD and QoL
60	*Urol Ann*	Asgari *et al.*	Iran	2013	149	Author-constructed questionnaire		70	16	63		Pros.	Global satisfaction was higher with CD and NB compared with IC. Continent UD provides better results in terms of QoL compared to IC
61	*Cent Eur J Urol*	Aboumarzouk *et al.*	Poland	2013	63	Assessment based on psychological, social, sexual and physical states (no particular scale used)		39		24		Pros.	No difference between the groups regarding QoL; no difference between either UD in all comparative aspects e.g. length of hospital stay, complications etc., except that the NB had a longer operative time
62	*Health Qual Life Outcomes*	Gacci *et al.*	Italy	2013	37	EORTC QLQ-C30	FACT-BL and QLQ-BLM30	16	12	9		Retro.	Patients with cutaneous ureterostomy had worse HRQoL compared to those who underwent IC or NB, primarily due to physical/emotional perception of body image.
63	*J Urol*	*Parekh* et al.	USA	2013	40							Pros.	
64	*Arch Esp Urol*	Fuentes *et al.*	Spain	2014	25	FACT-Bl		2	19	3		Retro.	Ureterosigmoidostomy may be a good choice for UD in selected patients, with similar QoL to other types of UD
65	*Urol Oncol*	Miyake *et al.*	Japan	2014	234	SF-36				234		Retro.	Both types resulted in satisfactory outcomes; sigmoid NB group appeared to be more favourable than ileal NB group in terms of long-term voiding function
66	*Ann Surg Oncol*	Rouanne *et al.*	France	2014	31	SF-12	Urinary symptom profile/Contilife questionnaire			31		Retro.	Ileal NB reconstruction provides long-term satisfaction with maintained HRQoL
67	*BJU Int*	Singh *et al.*	India	2014	164	EORTC QLQ-C30		80		84		Pros.	NB better QoL outcomes than IC
68	*Urology*	Large *et al.*	USA	2014	73	FACT-VCI		27		16		Pros.	Scores did not statistically differ from baseline to 6-month follow-up between UD types
69	*Urology*	Aboumohamed *et al.*	USA	2014	182	BCI	BIS	182				Retro.	RARC has comparable HRQoL outcomes to open RC; UD technique does not appear to affect QoL
70	*BJU Int*	Poch *et al.*	USA	2014	43	BCI	EORTC-BIS	38		5		?	HRQoL outcomes after RARC show recovery of urinary and bowel domains at ≤6 months
71	*Int J Urol*	Zahran *et al.*	Egypt	2014	74	EORTC QLQ-C30	FACT-Bl			74		Retro.	After ONB in women, HRQoL is lower than that of the normal population – night time incontinence being a particular issue
72	*World J Urol*	Mischinger *et al.*	Germany	2014	56	SF-36	QLQ-C30 + QLQ-BLM30 + TNQ			56		Pros.	Contradictory results – suggest that the questionnaires are not useful to evaluate HRQoL in patients with different NBs
73	*BJU Int*	Messer *et al.*	USA	2014	40	FACT-VCI		37		3		Pros.	HRQoL returns to baseline 3 months post-RC, with no signiﬁcant diﬀerence in HRQoL between open RC and RARC
74	*BMC Urol*	Huang *et al.*	China	2015	294	EORTC-QOL	BIS, BCI	78		39		Retro.	The mean BIS score in ileal ONB group patients was significantly better than that in IC group patients at the 1-year follow-up, but there was no significant difference at the long-term follow-up.
75	*Urol Oncol*	Goldberg *et al.*	Israel	2015	95	BCI		49		46		Retro.	Increased risk of urinary incontinence and sexual dysfunction for NB reconstruction vs IC
76	*Eur Urol*	Bochner *et al.*	USA	2015	124	Self-designed questionnaire	Global health, side effects, emotional	27 (r), 23 (o)	0 (r), 3(o)	33 (r) 32 (o)		Pros.	There were no clinical or statistical differences between the two arms in QoL change from baseline to 3 month or from 3 to 6 months in any of the evaluated domains
77	*Eur Urol*	Satkunasivam *et al.*	USA	2016	107	Modified BCI, SF-36	mucus- and pad-related questions included			28 (r), 79 (o)		Retro.	Ileal ONB had comparable bladder cancer-specific HRQOL scores to open ONB. However, pad size and daytime wetness were worse for ileal ONB, albeit over a significantly shorter follow-up
78	*BJU Int*	Longo *et al.*	Italy	2016	70	BCI – translated to Italian	Likert scale, BCI assessed stoma and appliance function	35	35			Retro.	Chronic ureteric stenting does not affect the QoL of patients with bladder cancer undergoing CD compared with those undergoing IC UD.
79	*Oncol Lett*	Liu *et al.*	China	2016	85	Karnofsky performance scale (functional), FACT-G, BSS		27	28 (traditional), 30 (tubeless)			Retro.	The HRQoL scores of the patients in the improved group were significantly higher than those of the patients in the other two groups, and the difference was statistically significant
80	*Eur Urol*	Khan *et al.*	UK	2016	164	FACT-Bl	BCa; Bladder Cancer Subscale	17 (o), 3 (r), 18 (l)		3 (o), 2 (r), 1 (l)		Pros.	There were no statistically significant relationships in QoL according to surgical arm (o, open; r, robotic; l, laparoscopic)
81	*J Urol*	Winters *et al.*	USA	2018	166							Retro.	
82	*Minerva Urol Nefrol*	Zahran *et al.*	Egypt	2017	145	EORTC-QLQ-C30 (translated to Arabic)	FACT-Bl	64		84		Retro.	In women, HRQoL is better after ONB than IC as long as continence status is preserved. If incontinence is expected, IC may be a better option for UD.
83	*Urology*	Gellhaus *et al.*	USA	2017	128	BCI		44		48	36 (IP)	Retro.	Urinary function but not urinary bother was significantly better in IC and IP compared to NB UDs. Older men with IC had better urinary function than older men with NB. In younger men, IP patients had significantly better urinary function than NB patients.
84	*Eur J Surg Oncol*	Mischinger *et al.*	Germany	2017	56	GIQLI					23 (Studer) 33(I-pouch)	Retro.	No significant differences in postoperative bowel disorders were found between both NB types
85	*World J Urol*	Kretschmer *et al.*	Germany	2017	121	EORTC–QLQ-C30 – German translation	ICIQ-SF questionnaire	50		50		Retro.	ONB is an independent predictor for better overall HRQoL at 3 months, but not 12 months after RC (global health score, physical functioning, role functioning)

CARE, Convalescence and Recovery Evaluation; GIQLI, Gastrointestinal Quality of Life Index: FLZM, Fragen zur Lebenszufriedenheit; HADS, Hospital Anxiety and Depression Scale; ICIQ-SF, International Consultation on Incontinence Questionnaire short form; IP, Indiana pouch; l, laparoscopic; MCT, meta-contrast technique; o, open; ONB, orthotopic NB; Pros., prospective; POMS, profile of mood status; r, robotic; Retro., retrospective; SWLS, Satisfaction With Life Scale; TNQ, neobladder-specific questionnaire; UUC, uretero-ureterocutaneostomy; VAS, visual analogue scale.

The overall proportion of reported IC patients reduced from 65% (784 patients) to 36% (899) and 35% (1664) from period-1 to period-3, whereas the proportion of reported NB patients increased from 4.5% (54) to 44% (1105) and 44% (2105). The reporting of both the UD types was broadly similar over the last two decades. Over the last few years there have also been QoL studies on laparoscopic and robot-assisted IC and NB UDs.

Overall, 43 (51%) studies came from Europe, 22 (26%) from the USA, and 16 (19%) from Asia (Table 3), with 16 studies published in *BJU International* (*British Journal of Urology* before 1999) and 10 studies in *Urology*.

**Table 3.** Geographical density and impact of studies over the last four decades.

Year	Number and country	Number and continent	Number and Journal
1980–1997	5 – Sweden 4 – Denmark 2 – UK, Germany 1 – USA, Japan, Norway	14 – Europe 1 – USA, Asia	5 – *Br J Urol (BJU Int)* 3 – *J Urol* 1 – *Eur Urol, Int J Urol*
1998–2007	7 – Japan 5 – USA 4 – Sweden 1 – Germany, Denmark, Netherlands, Canada, Austria, Turkey, Greece, Belgium	11 – Europe 7 – Asia 6 – USA	5 – *BJU Int* 3 – *Urology, J Urol* 2 – *Int J Urol, Eur Urol, Cancer*
2008-–2017	14 – USA 5 – Italy 4 – Germany 3 – UK, Japan, China 2 – Egypt 1 – Norway, Greece, Bosnia, South Korea, Canada, Iran, Poland, Spain, France, India, Israel	18 – Europe 15 – USA 8 – Asia 2 – Africa 1 – Middle East	7 – *Urology* 6 – *BJU Int* 3 – *Eur Urol, Eur J Surg Oncol* 2 – *World J Urol, Urol Oncol*

## Discussion

Over the last decade, there have been more QoL studies and more validated and prospective studies. Similarly, the proportionate numbers of NBs has also increased over the last two decades, with newer studies now reporting on laparoscopic and robot-assisted UDs (Tables 1 and 2). There has also been a rise in the number of studies reporting on QoL outcomes in these patients, demonstrating the importance placed on QoL in the last decade.

### Change in QoL trends over the last four decades

Whilst publication trends show that better reporting of QoL with more validated questionnaires are now being used, it seems that as long as the patient is well counselled and supported in their decision they learn to cope and adjust with their UD type [1].

Compared to previous decades, the past decade has seen an upsurge in focus on QoL outcomes in bladder cancer. This has occurred in tandem with the development of new and specific HRQoL instruments used in bladder cancer [1,8]. There has also been considerable variability in the use of QoL assessment tools, with a progressive uptake of validated assessment methods in the last decade. Our literature review revealed 13 of the 15 studies between 1987 and 1997 used a non-validated ad hoc (self-designed) instrument to assess QoL outcomes compared to only four in the 2009–2017 period. This suggests that the process of HRQoL measurement is becoming increasingly popular and perhaps clinically responsive.

The ad hoc instruments previously used were potentially poor measures of reliability and qualitative outcomes, and subject to bias due to their inherent non-validated nature [1,8]. In addition, there has been a gradual rise in the globalisation of quality assessment in patients after RC, given that 18 different countries are represented across the studies in 2009–2017 compared to only six in 1987–1997. Only one study across the last three decades accounted for sociocultural influences in health perception and QoL evaluation [11]. Perhaps there is a role for cross-cultural testing of these QoL instruments to ensure the validity and reliability of these tools across patients from other countries and cultures [12].

### Generic vs cancer-specific QoL assessment

In the past more generic QoL assessment tools were used. Previously, the Beck Depression Inventory (BDI) and Sickness Impact Profile (SIP) [13] were used to assess HRQoL across a wide range of medical conditions, and therefore these were not responsive to finer, qualitative, postoperative changes pertinent to bladder cancer and UD. Similarly, the 36-item short-form health survey (SF-36) [14], which was commonly used in this time period did not incorporate postoperative concerns specific to bladder surgery, including issues such as erectile dysfunction or urinary incontinence. Indeed, even cancer-specific scales [European Organisation for the Research and Treatment of Cancer Quality of Life 30-item core questionnaire (EORTC QLQ-30C) [15] and Functional Assessment of Cancer Therapy-General (FACT-G) [16]] failed to address specific domains of importance to patients with bladder cancer.

The importance of developing instruments that measure specific outcomes for patients with bladder cancer is slowly being addressed as demonstrated by the fact that 23 studies from 2009 onwards used a bladder-specific QoL tool. Tools such as the FACT-Bladder Cancer (FACT-BL), Bladder Cancer Index (BCI) [17], Body Image Scale (BIS), FACT-Vanderbilt Cystectomy Index (FACT-VCI) [18], suggest a greater appreciation for having a responsive tool that can identify specific concerns in post-RC patients and hopefully act as a framework to compare outcomes and validate more specific tools.

Despite several retrospective studies reporting no clear superiority for NB surgery [17,20–23], recent data suggest that NB is increasingly being offered to patients. Although the type of UD should be individualised, the surgeon or centre should be able to offer both types of UDs for surgical equipoise, based on patient preference.

Table 1 suggests a progressive increase in the number of prospective studies being performed in the last decade, along with a rise in the reported numbers of NB UDs. We can perhaps postulate that the shift towards NBs has predominantly been driven by improved surgical training in a more complex procedure and better patient counselling, offering a choice of UD rather than the QoL outcomes [24].

### Role of laparoscopic and robot-assisted surgery in post-RC QoL

The advent of minimally invasive surgery, such as robot-assisted RC (RARC) and laparoscopic surgery, has led to decreased length of stay and morbidity, and faster recovery. Multicentre data from the USA has suggested a significant rise in its use from 0.6% in 2004 to 12.8% in 2010 [2,3,25]. Poch et al. [26] assessed QoL before and after RARC and reported no significant QoL advantage for RARC. However, the authors found quicker return of urinary function and better body image postoperatively with intracorporeal vs extracorporeal UDs. Studies have failed to show a QoL benefit of RARC compared to open RC [2,27]. A recent meta-analysis also suggests postoperative HRQoL to be similar in patients undergoing RARC and open RC [28]. With the advent of enhanced recovery after surgery (ERAS) protocols, there is now a reduction in postoperative morbidity and hospital stay, with a recent study reporting higher emotional well-being in patients who underwent ERAS [29].

### Strengths and limitations of the review

Despite the current trend of QoL studies moving in the right direction with the increased use of validated and specific HRQoL measures; the fact remains that there are still significant challenges in measuring QoL in UD patients. Sexual dysfunction although common is perhaps poorly captured. Conversely, although disease-specific questionnaires are more responsive than generic questionnaires to subtle changes within disease-specific domains, the high disease-specific sensitivity of these questionnaires may limit the ability to account for unexpected events. For example, an unanticipated neurological adverse event may not be addressed in the disease-specific instrument’s questions and as a result this may not be reflected in an accurate QoL change. Of the newer QoL tools, the Bladder Utility Symptom Scale (BUSS) seems to be a novel patient-reported outcome instrument and measures HRQoL for all patients with bladder cancer regardless of treatment received or stage of the disease [30].

Various studies have been published investigating QoL after RC and UD. However, there is an extensive deal of heterogeneity amongst these studies with regards to methodology, the use of non-validated QoL instruments, and the underpowered and retrospective nature of the majority of data make interpretation difficult. Based on the present studies, QoL has not shown to be significantly variable across the different types of UD. As the majority of them are retrospective in nature, there also remains the risk of inherent selection bias. Furthermore, QoL is only measured postoperatively in most of these studies, and in the absence of preoperative QoL data, it is not truly possible to determine the effect of UD. With different approaches to UD and in the absence of any randomised trials, results from the ongoing prospective, multicentre, randomised trial of open vs robotic radical cystectomy (RAZOR) trial may provide an answer in the near future [31].

## Conclusion

The last four decades has seen gradual but significant improvements in the way QoL assessment is conducted in RC patients, with the implementation of several validated, bladder cancer-specific questionnaires and UD-specific constructs. The emergence of more prospective studies with validated QoL instruments has improved our ability to identify their QoL and to understand the differences between various UD types.

## Appendix 1 Reference list for the 85 included studies

[1] Jones MA, Breckman B, Hendry WF. Life with an ileal conduit: results of questionnaire surveys of patients and urological surgeons. *Br J Urol* 1980; 52: 21–5.

[2] Fosså SD, Reitan JB, Ous S, Kaalhus O. Life with an ileal conduit in cystectomized bladder cancer patients: expectations and experience. *Scand J Urol Nephrol* 1987; 21: 97–101.

[3] Boyd SD, Feinberg SM, Skinner DG, Lieskovsky G, Baron D, Richardson J. Quality of life survey of urinary diversion patients: comparison of ileal conduits versus continent Kock ileal reservoirs. *J Urol* 1987; 138: 1386–9. h

[4] Månsson A, Johnson G, Månsson W. Quality of life after cystectomy. Comparison between patients with conduit and those with continent caecal reservoir urinary diversion. *Br J Urol* 1988; 62: 240–5.

[5] Mommsen S, Jakobsen A, Sell A. Quality of life in patients with advanced bladder cancer. A randomized study comparing cystectomy and irradiation – the Danish Bladder Cancer Study Group (DAVECA protocol 8201). *Scand J Urol Nephrol* 1989; 125 (Suppl.): 115–20.

[6] Chadwick DJ, Stower MJ. Life with urostomy. *Br J Urol* 1990; 65: 189–91.

[7] Månsson A, Johnson G, Månsson W. Psychosocial adjustment to cystectomy for bladder carcinoma and effects on interpersonal relationships. *Scand J Caring Sci* 1991; 5: 129–34.

[8] Nordström GM, Nyman CR. Male and female sexual function and activity following ileal conduit urinary diversion. *Br J Urol* 1992; 70: 33–9.

[9] Nordström G, Nyman CR, Theorell T. Psychosocial adjustment and general state of health in patients with ileal conduit urinary diversion. *Scand J Urol Nephrol* 1992; 26: 139–47.

[10] Bjerre BD, Johansen C, Steven K. Health-related quality of life after urinary diversion: continent diversion with the Kock pouch compared with ileal conduit. A questionnaire study. *Scand J Urol Nephrol* 1994; 157 (Suppl.): 113–8.

[11] Bjerre BD, Johansen C, Steven K. Health-related quality of life after cystectomy: bladder substitution compared with ileal conduit diversion. A questionnaire survey. *Br J Urol* 1995; 75: 200–5.

[12] Gerharz EW, Weingartner K, Dopatka T, Kohl UN, Basler HD, Riedmiller HN. Quality of life after cystectomy and urinary diversion: results of a retrospective interdisciplinary study. *J Urol* 1997; 158: 778–85.

[13] Okada Y, Oishi K, Shichiri Y, Kakehi Y, Hamaguchi A, Tomoyoshi T et al. Quality of life survey of urinary diversion patients: comparison of continent urinary diversion versus ileal conduit. *Int J Urol* 1997; 4: 26–31.

[14] Filipas D, Egle UT, Büdenbender C, Fisch M, Fichtner J, Hoffmann SO et al. Quality of life and health in patients with urinary diversion: a comparison of incontinent versus continent urinary diversion. *Eur Urol* 1997; 32: 23–9.

[15] Bjerre BD, Johansen C, Steven K. A questionnaire study of sexological problems following urinary diversion in the female patient. *Scand J Urol Nephrol* 1997; 31: 155–60.

[16] Månsson A, Colleen S, Hermerén G, Johnson G. Which patients will benefit from psychosocial intervention after cystectomy for bladder cancer? *Br J Urol* 1997; 80: 50–7.

[17] Bjerre BD, Johansen C, Steven K. Sexological problems after cystectomy: bladder substitution compared with ileal conduit diversion. A questionnaire study of male patients. *Scand J Urol Nephrol* 1998; 32: 187–93.

[18] Weijerman PC, Schurmans JR, Hop WC, Schröder FH, Bosch JL. Morbidity and quality of life in patients with orthotopic and heterotopic continent urinary diversion. Urology 1998; 51: 51–56.

[19] Sullivan LD, Chow VD, Ko DS, Wright JE, McLoughlin MG. An evaluation of quality of life in patients with continent urinary diversions after cystectomy. *Br J Urol* 1998; 81: 699–704.

[20] Månsson A, Christensson P, Johnson G, Colleen S. Can preoperative psychological defensive strategies, mood and type of lower urinary tract reconstruction predict psychosocial adjustment after cystectomy in patients with bladder cancer? *Br J Urol* 1998; 82: 348–56.

[21] Hart S, Skinner EC, Meyerowitz BE, Boyd S, Lieskovsky G, Skinner DG. Quality of life after radical cystectomy for bladder cancer in patients with an ileal conduit, cutaneous or urethral kock pouch. *J Urol* 1999; 162: 77–81.

[22] Kitamura H, Miyao N, Yanase M, Masumori N, Matsukawa M, Takahashi A et al. Quality of life in patients having an ileal conduit, continent reservoir or orthotopic neobladder after cystectomy for bladder carcinoma. *Int J Urol* 1999; 6: 393–9.

[23] Hardt J, Filipas D, Hohenfellner R, Egle UT. Quality of life in patients with bladder carcinoma after cystectomy: first results of a prospective study. *Qual Life Re*s 2000; 9: 1–12.

[24] McGuire MS, Grimaldi G, Grotas J, Russo P. The type of urinary diversion after radical cystectomy significantly impacts on the patient’s quality of life. *Ann Surg Oncol* 2000; 7: 4–8.

[25] Fujisawa M, Isotani S, Gotoh A, Okada H, Arakawa S, Kamidono S. Health-related quality of life with orthotopic neobladder versus ileal conduit according to the SF-36 survey. *Urology* 2000; 55: 862–5.

[26] Hobisch A, Tosun K, Kinzl J, Kemmler G, Bartsch G, Höltl L et al. Quality of life after cystectomy and orthotopic neobladder versus ileal conduit urinary diversion. *World J Urol* 2000; 18: 338–44.

[27] Kulaksizoglu H, Toktas G, Kulaksizoglu IB, Aglamis E, Unlüer E. When should quality of life be measured after radical cystectomy? *Eur Urol* 2002; 42: 350–5.

[28] Månsson Å, Davidsson T, Hunt S, Månsson W, The quality of life in men after radical cystectomy with a continent cutaneous diversion or orthotopic bladder substitution: is there a difference? *BJU Int* 2002; 90: 386–90.

[29] Hara I, Miyake H, Hara S, Gotoh A, Nakamura I, Okada H et al. Health-related quality of life after radical cystectomy for bladder cancer: a comparison of ileal conduit and orthotopic bladder replacement. *BJU Int* 2002; 89: 10–3.

[30] Dutta SC, Chang SC, Coffey CS, Smith JA, Jack G, Cookson MS. Health related quality of life assessment after radical cystectomy: comparison of ileal conduit with continent orthotopic neobladder. *J Urol* 2002; 168: 164–7.

[31] Henningsohn L, Wijkström H, Steven K, Pedersen J, Ahlstrand C, Aus G et al. Relative importance of sources of symptom-induced distress in urinary bladder cancer survivors. *Eur Urol* 2003; 43: 651–62.

[32] Protogerou V, Moschou M, Antoniou N, Varkarakis J, Bamias A, Deliveliotis C. Modified S-pouch neobladder vs ileal conduit and a matched control population: a quality-of-life survey. *BJU Int* 2004; 94: 350–4.

[33] Joniau S, Benijts J, Van Kampen M, De Waele M, Ooms J, Van Cleynenbreugel B et al. Clinical experience with the n-shaped ileal neobladder: assessment of complications, voiding patterns, and quality of life in our series of 58 patients. *Eur Urol* 2005; 47: 666–73.

[34] Yoneda T, Adachi H, Urakami S, Kishi H, Shigeno K, Shiina H et al. Health related quality of life after orthotopic neobladder construction and its comparison with normative values in the Japanese population. *J Urol* 2005; 174: 1944–7.

[35] Allareddy V, Kennedy J, West MM, Konety BR. Quality of life in long-term survivors of bladder cancer. *Cancer* 2006; 106: 2355–62.

[36] Kikuchi E, Horiguchi Y, Nakashima J, Ohigashi T, Oya M, Nakagawa K et al. Assessment of long-term quality of life using the fact-bl questionnaire in patients with an ileal conduit, continent reservoir, or orthotopic neobladder. *Jpn J Clin Oncol* 2006; 36: 712–6.

[37] Harano M, Eto M, Nakamura M, Hasegawa Y, Kano M, Yamaguchi A et al. A pilot study of the assessment of the quality of life, functional results, and complications in patients with an ileal neobladder for invasive bladder cancer. *Int J Urol* 2007; 14: 112–7.

[38] Gilbert SM, Wood DP, Dunn RL, Weizer AZ, Lee CT, Montie JE et al. Measuring health-related quality of life outcomes in bladder cancer patients using the Bladder Cancer Index (BCI). *Cancer* 2007; 109: 1756–62.

[39] Saika T, Arata R, Tsushima T, Nasu Y, Suyama B, Takeda K et al. Health-related quality of life after radical cystectomy for bladder cancer in elderly patients with an ileal conduit, ureterocutaneostomy, or orthotopic urinary reservoir: a comparative questionnaire survey. *Acta Med Okayama* 2007; 61: 199–203.

[40] Månsson Å, Al Amin M, Malmström PU, Wijkström H, Abol Enein H, Månsson W. Patient-assessed outcomes in Swedish and Egyptian men undergoing radical cystectomy and orthotopic bladder substitution – a prospective comparative study. *Urology* 2007; 70: 1086–90.

[41] Autorino R, Quarto G, Di Lorenzo G, De Sio M, Perdonà S, Giannarini G et al. Health related quality of life after radical cystectomy: comparison of ileal conduit to continent orthotopic neobladder. *Eur J Surg Oncol* 2009; 35: 858–64.

[42] Sogni F, Brausi M, Frea B, Martinengo C, Faggiano F, Tizzani A et al. Morbidity and quality of life in elderly patients receiving ileal conduit or orthotopic neobladder after radical cystectomy for invasive bladder cancer. *Urology* 2008; 71: 919–23.

[43] Yuh B, Butt Z, Fazili A, Piacente P, Tan W, Wilding G et al. Short-term quality-of-life assessed after robot-assisted radical cystectomy: a prospective analysis. *BJU Int* 2009: 103: 800–4.

[44] Frich PS, Kvestad CA, Angelsen A. Outcome and quality of life in patients operated on with radical cystectomy and three different urinary diversion techniques. *Scand J Urol Nephrol* 2009; 43: 37–41.

[45] Philip J, Manikandan R, Venugopal S, Desouza J, Javlé PM. Orthotopic neobladder versus ileal conduit urinary diversion after cystectomy – a quality-of-life based comparison. *Ann R Coll Surg Engl* 2009; 91: 565–9.

[46] Somani BK, Gimlin D, Fayers P, N’Dow J. Quality of life and body image for bladder cancer patients undergoing radical cystectomy and urinary diversion – a prospective cohort study with a systematic review of literature. *Urology* 2009; 74: 1138–43.

[47] Miyake H, Furukawa J, Muramaki M, Takenaka A, Fujisawa M. Orthotopic sigmoid neobladder after radical cystectomy: assessment of complications, functional outcomes and quality of life in 82 Japanese patients. *BJU Int* 2010; 106: 412–6.

[48] Large MC, Katz MH, Shikanov S, Eggener SE, Steinberg GD. Orthotopic neobladder versus Indiana pouch in women: a comparison of health related quality of life outcomes. *J Urol* 2010; 183: 201–6.

[49] Hedgepeth RC, Gilbert SM, He C, Lee CT, Wood DP. Body image and bladder cancer specific quality of life in patients with ileal conduit and neobladder urinary diversions. *Urology* 2010; 76: 671–5.

[50] Vakalopoulos I, Dimitriadis G, Anastasiadis A, Gkotsos G, Radopoulos D. Does intubated uretero-ureterocutaneostomy provide better health-related quality of life than orthotopic neobladder in patients after radical cystectomy for invasive bladder cancer? *Int Urol Nephrol* 2011; 43: 743–8.

[51] Erber B, Schrader M, Miller K, Schostak M, Baumunk D, Lingnau A et al. Morbidity and quality of life in bladder cancer patients following cystectomy and urinary diversion: a single-institution comparison of ileal conduit versus orthotopic neobladder. *ISRN Urol* 2012: 2012: 342,796. DOI:10.5402/2012/342,796.

[52] Anderson CB, Feurer ID, Large MC, Steinberg GD, Barocas DA, Cookson MS et al. Psychometric characteristics of a condition-specific, health-related quality-of-life survey: the FACT-Vanderbilt Cystectomy Index. *Urology* 2012; 80: 77–83.

[53] Miyake H, Furukawa J, Muramaki M, Inoue T, Fujisawa M. Health related quality of life after radical cystectomy: Comparative study between orthotopic sigmoid versus ileal neobladders. *Eur J Surg Oncol* 2012; 38: 1089–94.

[54] Stegemann A, Rehman S, Brewer K, Kesavadas T, Hussain A, Chandrasekhar R et al. Short-term patient-reported quality of life after robot-assisted radical cystectomy using the convalescence and recovery evaluation. *Urology* 2012; 79: 1274–80.

[55] Mucciardi G, Macchione L, Galì A, di Benedetto A, Subba E, Pappalardo R et al. [Quality of life and overall survival in high risk patients after radical cystectomy with a simple urinary derivation]. Cir Esp 2015; 93: 368–74.

[56] Prcic A, Aganovic D, Hadziosmanovic O. Sickness Impact Profile (SIP) score, a good alternative instrument for measuring quality of life in patients with ileal urinary diversions. Acta Inform Med 2013; 21: 160–5.

[57] Shim B, Kim KH, Yoon H, Park YY, Lee DH. Body image following radical cystectomy and ileal neobladder or conduit in korean patients. *Korean J Urol* 2014; 55: 161–6.

[58] Yang M, Wang H, Wang J, Ruan M. Impact of invasive bladder cancer and orthotopic urinary diversion on general health-related quality of life: An SF-36 survey. *Mol Clin Oncol* 2013; 1: 758–62.

[59] Metcalfe M, Estey E, Jacobsen NE, Voaklander D, Fairey AS. Association between urinary diversion and quality of life after radical cystectomy. *Can J Urol* 2013; 20: 6626–31.

[60] Asgari MA, Safarinejad MR, Shakhssalim N, Soleimani M, Shahabi A, Amini E. Quality of life after radical cystectomy for bladder cancer in men with an ileal conduit or continent urinary diversion: A comparative study. *Urol Ann* 2013; 5: 190–6.

[61] Aboumarzouk OM, Drewa T, Olejniczak P, Chlosta PL. Laparoscopic radical cystectomy: neobladder or ileal conduit, debate still goes on. *Cent Eur J Urol* 2014; 67: 9–15.

[62] Gacci M, Saleh O, Cai T, Gore JL, D’Elia C, Minervini A et al. Quality of life in women undergoing urinary diversion for bladder cancer: results of a multicenter study among long-term disease-free survivors. *Health Qual Life Outcomes* 2013; 11: 43. DOI:10.1186/1477–7525-11–43.

[63] Parekh DJ, Messer J, Fitzgerald J, Ercole B, Svatek R. Perioperative outcomes and oncologic efficacy from a pilot prospective randomized clinical trial of open versus robotic assisted radical cystectomy. *J Urol* 2013; 189: 474–9.

[64] Fuentes J, Ramos E, Truan D, Portillo JA, Campos-Juanatey F, Gala L et al. Review of a series of cystectomies in women for bladder cancer: complications and quality of life. *Arch Esp Urol* 2014; 67: 303–12.

[65] Miyake H, Furukawa J, Sakai I, Muramaki M, Yamashita M, Inoue TA et al. Orthotopic sigmoid vs. ileal neobladders in Japanese patients: a comparative assessment of complications, functional outcomes, and quality of life. *Urol Oncol* 2013; 31: 1155–60.

[66] Rouanne M, Legrand G, Neuzillet Y, Ghoneim T, Cour F, Letang N et al. Long-term women-reported quality of life after radical cystectomy and orthotopic ileal neobladder reconstruction. *Ann Surg Oncol* 2014; 21: 1398–404.

[67] Singh V, Yadav R, Sinha RJ, Gupta DK. Prospective comparison of quality-of-life outcomes between ileal conduit urinary diversion and orthotopic neobladder reconstruction after radical cystectomy: a statistical model. *BJU Int* 2014; 113: 726–32.

[68] Large MC, Malik R, Cohn JA, Richards KA, Ganshert C, Kunnavakkum R et al. Prospective health-related quality of life analysis for patients undergoing radical cystectomy and urinary diversion. *Urology* 2014; 84: 808–14.

[69] Aboumohamed AA, Raza SJ, Al-Daghmin A, Tallman C, Creighton T, Crossley H et al. Health-related quality of life outcomes after robot-assisted and open radical cystectomy using a validated bladder-specific instrument: a multi-institutional study. *Urology* 2014; 83: 1300–8.

[70] Poch MA, Stegemann AP, Rehman S, Sharif MA, Hussain A, Consiglio JD et al. Short-term patient reported health-related quality of life (HRQL) outcomes after robot-assisted radical cystectomy (RARC). *BJU Int* 2014; 113: 260–5.

[71] Zahran MH, El-Hefnawy AS, Zidan EM, El-Bilsha MA, Taha DE, Ali-El-Dein B. Health-related quality of life after radical cystectomy and neobladder reconstruction in women: Impact of voiding and continence status. *Int J Urol* 2014; 21: 887–92.

[72] Mischinger J, Abdelhafez MF, Todenhöfer T, Schwentner C, Aufderklamm S, Stenzl A et al. Quality of life outcomes after radical cystectomy: long-term standardized assessment of Studer Pouch versus I-Pouch. *World J Urol* 2015; 33: 1381–7.

[73] Messer JC, Punnen S, Fitzgerald J, Svatek R, Parekh DJ. Health-related quality of life from a prospective randomised clinical trial of robot-assisted laparoscopic vs open radical cystectomy. *BJU Int* 2014; 114: 896–902.

[74] Huang Y, Pan X, Zhou Q, Huang H, Li L, Cui X et al. Quality-of-life outcomes and unmet needs between ileal conduit and orthotopic ileal neobladder after radical cystectomy in a Chinese population: a 2-to-1 matched-pair analysis. *BMC Urol* 2015; 15: 117. DOI:10.1186/s12894-015–0113-7.

[75] Goldberg H, Baniel J, Mano R, Rotlevy G, Kedar D, Yossepowitch O. Orthotopic neobladder vs. ileal conduit urinary diversion: A long-term quality-of-life comparison. *Urol Oncol* 2016; 34: 121.e1-121.e7. DOI:10.1016/j.urolonc.2015.10.006.

[76] Bochner BH, Dalbagni G, Sjoberg DD, Silberstein J, Keren Paz GE, Donat SM et al. Comparing open radical cystectomy and robot-assisted laparoscopic radical cystectomy: A randomized clinical trial. *Eur Urol* 2015; 67: 1042–50.

[77] Satkunasivam R, Santomauro M, Chopra S, Plotner E, Cai J, Miranda G et al. Robotic intracorporeal orthotopic neobladder: urodynamic outcomes, urinary function, and health-related quality of life. *Eur Urol* 2016; 69: 247–53.

[78] Longo N, Imbimbo C, Fusco F, Ficarra V, Mangiapia F, Di Lorenzo G et al. Complications and quality of life in elderly patients with several comorbidities undergoing cutaneous ureterostomy with single stoma or ileal conduit after radical cystectomy. *BJU Int* 2016; 118: 521–6.

[79] Liu Z, Tian Q, Xia S, Yin H, Yao D, Xiu Y. Evaluation of the improved tubeless cutaneous ureterostomy technique following radical cystectomy in cases of invasive bladder cancer complicated by peritoneal metastasis. *Oncol Lett* 2016; 11: 1401–5.

[80] Khan MS, Gan C, Ahmed K, Ismail AF, Watkins J, Summers JA et al. A single-centre early phase randomised controlled three-arm trial of open, robotic, and laparoscopic radical cystectomy (CORAL). *Eur Urol* 2016; 69: 613–21.

[81] Winters BR, Wright JL, Holt SK, Dare A, Gore JL, Schade GR. Health related quality of life following radical cystectomy: compariative analysis from medicare health outcomes survey. *J Urol* 2018; 199: 669–75.

[82] Zahran MH, Taha DE, Harraz AM, Zidan EM, El-Bilsha MA, Tharwat M et al. Health related quality of life after radical cystectomy in women: Orthotopic neobladder versus ileal loop conduit and impact of incontinence. *Minerva Urol Nefrol* 2017; 69: 262–70.

[83] Gellhaus PT, Cary C, Kaimakliotis HZ, Johnson CS, Weiner M, Koch MO et al. Long-term health-related quality of life outcomes following radical cystectomy. *Urology* 2017; 106: 82–6.

[84] Mischinger J, Abdelhafez MF, Rausch S, Todenhöfer T, Neumann E, Aufderklamm S et al. Perioperative morbidity, bowel function and oncologic outcome after radical cystectomy and ileal orthotopic neobladder reconstruction: Studer-pouch versus I-pouch. *Eur J Surg Oncol* 2018; 44: 178–84.

[85] Kretschmer A, Grimm T, Buchner A, Grabbert M, Jokisch F, Schneevoigt BS et al. Prospective evaluation of health-related quality of life after radical cystectomy: focus on peri- and postoperative complications. *World J Urol* 2017; 35: 1223–31.
